# Remote buprenorphine-naloxone initiation as an essential service for people with chronic pain and opioid dependence during the COVID-19 pandemic: Case reports, clinical pathways, and implications for the future

**DOI:** 10.1080/24740527.2020.1795634

**Published:** 2020-09-15

**Authors:** Hance Clarke, Aliza Weinrib, Yuvaraj Kotteeswaran, Joel Katz, Alvis Yu, Robert Tanguay

**Affiliations:** a Department of Anesthesiology and Pain Medicine, University of Toronto, Toronto, Ontario, Canada; b Department of Anesthesia and Pain Management, Toronto General Hospital, University Health Network, Toronto, Ontario, Canada; c University of Toronto Centre for the Study of Pain, Toronto, Ontario, Canada; d Department of Psychology, York University, Toronto, Ontario, Canada; e Department of Family Medicine, University of Calgary, Calgary, Alberta, Canada; f Opioid Deprescribing Program, Alberta Health Services, Calgary, Alberta, Canada; g Department of Psychiatry and Surgery, University of Calgary, Calgary, Alberta, Canada; h Hotchkiss Brain Institute, Cumming School of Medicine, University of Calgary, Calgary, Alberta, Canada

**Keywords:** chronic pain, suboxone, COVID-19, telemedicine, buprenorphine-naloxone, telepsychology

## Abstract

Many health care professions have reacted swiftly to the COVID-19 pandemic. In-person care has been ramped down and telemedicine/telehealth has been thrust to the forefront of clinical care. For people living with chronic pain and often concomitantly dealing with opioid-related issues, this is a time of great stress. With population-wide movements to shelter in place, people living with pain are more isolated, more stressed, and more vulnerable to mental health concerns like depression and anxiety that can increase pain-related suffering. This article presents two case reports of patients struggling with chronic pain and opioid dependence in which a telemedicine-based buprenorphine-naloxone conversion was chosen as a treatment option by two Canadian programs: The Transitional Pain Service at the Toronto General Hospital in Toronto, Ontario, and The Opioid Deprescribing Program in Calgary, Alberta. Both cases presented highlight the use of telemedicine during the COVID-19 pandemic and suggest that there will be substantial need for these services well beyond the apex of the crisis. A buprenorphine-naloxone home induction protocol is presented and we provide insight into important lessons learned regarding the appropriate selection of patients with chronic pain struggling with opioid use disorder for buprenorphine-naloxone conversion. The provision of health care during the COVID-19 pandemic has rapidly forced practitioners to evolve novel health care practices, and these changes will have long-term implications.

## RÉSUMÉ

De nombreuses professions de santé ont réagi rapidement à la pandémie de COVID-19. Les soins en personne ont diminué, tandis que la télémédecine et la télésanté ont été propulsées au premier plan des soins cliniques. Pour les personnes vivant avec la douleur chronique, souvent confrontées de manière concomitante à des problèmes liés aux opioïdes, il s’agit d’une période de grand stress. Avec les mouvements de confinemen de la population mis en place, les personnes vivant avec la douleur sont plus isolées, plus stressées et plus vulnérables aux problèmes de santé mentale comme la depression et l’anxiété, qui peuvent augmenter la souffrance liée à la douleur. Cet article présente deux rapports de cas de patients aux prises avec la douleur chronique et la dépendance aux opioides où la conversion à la buprénorphine-naloxone par télémédecine a été choisie comme option de traitement par deux programmes canadiens : Le Service de la douleur transitoire de l’Hôpital général de Toronto, en Ontario, et le Programme de déprescription des opioides à Calgary, Alberta. Les deux cas présentés mettent en évidence l’utilisation de la télémédecine pendant la pandémie de COVID-19 et indiquent qu’il y aura un besoin important pour ces services bien au-delà du sommet de la crise. Un protocole d’induction de la buprénorphine-naloxone à domicile est présenté et nous donnons un aperçu des seignemens tirés quant à la selection appropriée de patients souffrant de douleur chronique et d’un trouble lié à l’usage d’opioïdes pour la conversion à la buprénorphine-naloxone. La prestation de soins de santé durant la pandémie de COVID-19 a rapidement obligé les praticiens à mettre au point de nouvelles pratiques de soins de santé, et ces changements auront des implications à long terme.

## Introduction

In response to the COVID-19 pandemic, the health care world has reacted swiftly. Most health care resources have been redirected with the overarching goal of protecting society, while ramping down in-person care in the hopes of “flattening the curve” and stopping the rapid spread of this deadly virus. Consequently, patients are enduring significant delays to their care pathways, having had elective procedures—as well as potentially life-saving procedures, such as cancer surgeries—postponed for weeks and potentially months. The debate over which patient visits/procedures are considered elective vs. essential changes daily. Practice guidelines are urgently needed, and experts have begun to publish treatment recommendations—for example, an expert panel of pain physicians, psychologists, and researchers from Europe and North America recently published chronic pain practice recommendations to guide treatment during COVID-19.^1^

For people living with chronic pain and often concomitantly dealing with opioid-related issues,^2^ this is a time of great stress. With population-wide movements to shelter in place, people living with pain are more isolated, more stressed, and more vulnerable to mental health concerns like depression and anxiety that can increase pain-related suffering. In addition, patients do not have access to in-person services like physiotherapy and some interventional procedures during this time. With ongoing global supply chain issues,^3^ the fear of running out of medications is significant among patients consuming prescription opioid medications. Long-acting opioid formulations are on back order without a clear indication of when that might be remedied. This shortage can have severe consequences, both physical and psychosocial. Some patients may suffer in isolation as they cope with significant opioid-related withdrawal; others may not be able to tolerate this distress and turn to high-risk illicit opioids in an attempt to mitigate their withdrawal symptoms and distress.^4^

For people living with chronic pain who take prescription opioid medications, health care delivery is an *essential* service. Physicians’ offices cannot simply close and abandon their patients. Interdisciplinary pain treatment must instead be triaged into levels of care with clear indications for in-person and remote/virtual intervention. Services that must be delivered face-to-face (e.g., high-priority interventional procedures) can be prioritized for in-person delivery, and clinical care that can be conducted remotely should be adapted to virtual delivery in order to reduce risk of COVID-19 transmission for patients and providers alike. The risk–benefit analysis for in-person care has been abruptly turned on its head. Accordingly, a key question for clinicians now is how clinical pathways can be effectively adapted for telemedicine and virtual health care. Over the past several years, telemedicine had been slowly progressing as a means of reaching rural clientele^5^; however, now its role must rapidly expand to reach patients who are practicing physical distancing to prevent the spread of COVID-19.

In addition to practice-related questions, this rapidly emerging new paradigm raises questions about what technological means should be used by health care providers to communicate remotely with patients, as well as remotely with one another. Health care systems around the world are being thrust into utilizing existing telephone infrastructure and web-based video conferencing platforms, including those developed by public health institutions (e.g., Ontario Telehealth Network) and those developed commercially (e.g., Zoom).

This article describes care pathways for buprenorphine-naloxone induction when in-person care is not the best option. Two patient case reports will be presented, the first treated by the Transitional Pain Service (TPS) at the Toronto General Hospital. This began with an in-person visit prior to the COVID-19 shut down and rapidly turned into a case needing an interdisciplinary health care model with virtual visits. The second case is an example of a home induction of buprenorphine-naloxone in a person living with chronic pain during the COVID-19 shutdown illustrating an established care pathway developed by the team at the Opioid Deprescribing Program in Calgary. The initials used throughout the article are pseudonyms to protect the patients’ identities. Practice issues and lessons learned will be discussed, with the hope that the home induction protocol we present may continue to be used once the current pandemic has ended. A written informed consent form was signed by each patient who agreed to the publication of the present case report and who also reviewed the article and approved the details of their case.

## Case #1: Transition to Buprenorphine-naloxone during the COVID-19 Crisis in the Transitional Pain Service, Toronto General Hospital, Toronto, Ontario

C.D. is a 67-year-old woman who had to reduce her work commitments as of 2014 due to pain-related disability after developing postherpetic neuralgia (PHN) of her face and head. On presentation to the TPS clinic at the start of the COVID-19 pandemic in early March 2020, her pain complaint was mainly on the right side of the head in the cranial nerve V1 distribution of the trigeminal nerve. Her pain was most often in the severe range, with the lowest pain severity score in the moderate range at 6/10 on the 0–10 pain intensity numeric rating scale. Previous visits with neurologists had ruled out migraines or trigeminal neuralgia.

Over the years, C.D. had tried gabapentin to a maximum dose of 3600 mg/day, pregabalin without convincing results, levetiracetam (intolerant due to adverse effects), and carbamazepine 900 mg/day with little benefit. A recent trial of 0.5 g/day of physician-authorized THC-dominant cannabis via vaping worsened her symptoms and caused paranoid hallucinations. CBD oil 20 mg/ml 0.5 ml TID also did not improve her symptoms. A neurosurgeon had suggested the possibility of peripheral neurostimulation; however, because results were often mixed, C.D. decided against it. At the time of the initial assessment, C.D. was taking 60 mg of oxycodone daily, which she would supplement with a carbamazepine tablet (100 mg) on the occasional day when her pain was excruciating. Accordingly, her morphine equivalent dose at TPS intake was 90 mg/day.

C.D. reported that she wished to stop using oxycodone, in part because it was providing only 10% to 20% pain relief and in part because she was concerned about dependence, but she could not taper the oxycodone (even very slowly) because it would induce vomiting. Indeed, at the initial visit to the TPS she kept a container close by in case she needed to vomit, which she did intermittently. It appeared that in addition to severe PHN in the cranial nerve V1 distribution, she had developed an opioid-induced cyclical vomiting syndrome^6^ in which vomiting is triggered by either opioid tapering or consuming oxycodone tablets. Treatment options were discussed, including rotation to another opioid or conversion to buprenorphine-naloxone. The shared decision was made to initiate the transition to buprenorphine-naloxone. The plan was that once she was stable on a dose of buprenorphine-naloxone, we could target a multimodal plan for her PHN symptoms.

### Suboxone Induction

Given our experience with previous inductions, we selectively chose to start patients on a buprenorphine transdermal patch prior to a buprenorphine-naloxone induction to mitigate some of the anticipated adverse induction withdrawal symptoms, in particular patients consuming less than 90 mg/day. Two 20 μg/h buprenorphine patches (40 μg/h) were placed on her arm 3 days prior to the buprenorphine-naloxone induction. The rationale for this treatment stemmed from H.C.’s experience with a patient who did not need to take an oral buprenorphine-naloxone tablet on the day of the planned induction when converting from low-dose oxycontin/oxycodone (i.e., 60 mg/day) with placement of a transdermal buprenorphine patch. C.D. was advised to stop taking oxycodone at 8 p.m. the night before (12 h prior to the planned induction). On the morning of the induction, C.D. presented to the TPS clinic with a Clinical Opioid Withdrawal Score (COWS)^7^ of 5. By midday her COWS score was 9 and she displayed increasing restlessness and distress. Y.K. then initiated the first 2 mg dose of sublingual (SL) buprenorphine-naloxone. By 5 p.m. that afternoon (after one of the buprenorphine 20 μg patches had been removed), her COWS score had increased to 14. She was given another 4 mg of buprenorphine-naloxone, discharged home on the remaining 20 μg/h buprenorphine patch, and given instructions to take additional 2 mg tablets of buprenorphine/naloxone as needed overnight to manage her withdrawal symptoms up to a maximum of 16 mg.

The introduction of the buphrenorphine patch prior to the initiation of oral buprenorphine-naloxone for C.D. is an example of a microdosing technique. Traditional induction of buprenorphine-naloxone requires patients to be in mild to moderate withdrawal from other opioids before administration of buprenorphine-naloxone. This period of opioid withdrawal can be poorly tolerated by patients and may discourage induction, improving retention in people with opioid use disorder. Furthermore, tapering of the full agonist without replacement with partial μ-opioid agonist bears the risk of relapse to illicit opioid use. The Bernese method describes a microdosing induction regimen for buprenorphine that overcomes this opioid withdrawal effect.^8^ Microdosing can be done either by the sublingual route^8^ or the transdermal route presented in our case report.^9,10^ Recently, rapid induction of microdosing by the sublingual route has become more common.^11,12^ The advantage of the rapid induction microdosing is that it takes 3 to 5 days, in comparison to the Bernese method, which usually takes more than 10 days. The microdosing regimens explained in the above literature tend to occur in classic inpatient settings. However, it has become clear that the microdosing induction technique is manageable and effective in an outpatient setting. A less frequent dosing regimen may be more practical for outpatients.

Our typical clinical care pathway would have been to see C.D. in person, in clinic, the following morning. However, given the rapidly escalating concern over COVID-19, the hospital mandated that all face-to-face patient visits to the clinic be canceled and clinical staff transition to seeing patients using telehealth services.

C.D. was called the following morning to inform her of the unanticipated COVID-19 mandate and to monitor her progress. C.D. reported that the induction had not progressed smoothly and that she was in significant distress. Several hours after leaving the TPS clinic the previous day, she presented to her local emergency department. The emergency physician astutely ascertained that she was dealing with heightened opioid withdrawal, gave her another 2 mg tablet of buprenorphine-naloxone, and discharged her home to continue the induction according to our previous instructions. She had taken another 2 mg buprenorphine-naloxone overnight but during H.C.’s telephone call with her in the morning she continued vomiting throughout the conversation, so it was decided that C.D. would take the entire 12 mg dose that morning. A requisition for a prescription of 12 mg/day SL buprenorphine-naloxone was sent to her pharmacy (1 × 8 mg/2 mg and 2 × 2 mg/0.5 mg tablets). A follow-up call was made that day to C.D. at 12 p.m., by which time she reported that she was starting to feel better. Later that evening as withdrawal symptoms intensified, C.D. took another 2 mg buprenorphine-naloxone. Given the rocky nature of this induction, the hope was that she was over the worst and was heading toward a smooth zone moving forward.

On the third morning after the start of the buprenorphine-naloxone induction, around 9 a.m., H.C. received a distressed mobile communication from C.D. She explained that she was not able to tolerate the large 8 mg tablet. It caused her to gag when placed under the tongue; hence, she had spit out her morning dose. During the telephone assessment, it was unclear how much of the dose she had absorbed. She stated that she was trembling and alone (her partner was an essential worker who was attending to his public duties), vomiting continually, and unable to leave her bed. At this point, and in the midst of COVID-19, H.C. was struggling to provide the support C.D. required and enlisted the help of the TPS multidisciplinary team (Y.K. and A.W.), who were both in self-isolation, as well as his colleague (R.T.), who resides in Alberta. These colleagues agreed to an impromptu teleconference meeting to discuss the challenges of the current case. Following the meeting, H.C. called C.D. to present a path forward that included a modified pharmacological approach and psychological intervention with A.W.

On the call to C.D., she reported that the day before, she had left her buprenorphine-naloxone tablets in a downstairs location in her house and was currently unable to navigate the stairs to retrieve them given how weak and unstable she felt. H.C. and C.D. devised a plan for one of C.D.’s neighbors to help by bringing C.D.’s 2 mg tablets from the downstairs location to the upper floor, leaving the medication outside C.D.’s bedroom door. Once her neighbor had departed, C.D. would retrieve the tablets. This plan, for the most part, maintained the physical distancing polices that the provincial government had implemented.

Because C.D. was not able to take an 8 mg tablet, the team’s plan was to titrate to 16 mg buprenorphine-naloxone, in 2 mg dose escalations, from the 14 mg dose the day before. Given the uncertainty of how much of the 8 mg tablet had been absorbed in the morning, H.C. instructed C.D. to start afresh and take 5 × 2 mg tablets over the next hour with a goal to consume three more 2 mg tablets prior to bedtime for a total daily dose of 16 mg. A telephone call changing her prescriptions from 8 mg to 2 mg tablets of buprenorphine-naloxone was made to the pharmacist who facilitated this change. During the COVID-19 pandemic, Health Canada issued a temporary exemption for patients, practitioners, and pharmacists prescribing and providing controlled substances that enables telephone orders.^13^

H.C. also informed C.D. that A.W. had agreed to provide her urgent psychological intervention sessions to help her get through this extremely distressing period. That Friday afternoon A.W. reached out to C.D., and by the following morning C.D.’s vomiting had subsided for the first time in weeks. Over the course of the weekend, C.D. reported that she was able to get out of the house (she lives in a rural area of the province), tend to her horses, and walk her dog, significant achievements relative to the pre-induction and immediate postinduction periods.

### One week Postinduction

C.D. was stable on 16 mg buprenorphine-naloxone a day after the oxycodone to buprenorphine-naloxone conversion, and there was complete resolution of her nausea and vomiting symptoms. However, she reported that she was now plagued by an enormous amount of anxiety, with panic attacks four to five times a day. One of the adverse effects associated with buprenorphine-naloxone is significant anxiety, which may be related to an increase in limbic tone. Given her significant emotional distress, a shared decision was again made to reduce the buprenorphine-naloxone dose to 14 mg/day with the goal of getting the dose down to 8 mg, at which point her anxiety might be reduced and the focus would shift to starting neuropathic pain medications for her PHN. Unfortunately, by the following morning, the vomiting cycle had resumed, and it was apparent that C.D. was exquisitely sensitive to changes to her opioid dose. C.D. once again called upon the services of A.W. and we immediately reinstated the 16 mg/day dose.

### Implementing Psychological Care Via Telehealth

For people living with pain and opioid dependence, psychological care during buprenorphine-naloxone induction generally is brief (e.g., two to three sessions). In terms of treatment techniques, sessions are modeled on the focused acceptance and commitment therapy (FACT) protocols developed by Kirk Strosahl and colleagues for use with medical patients.^14^ In addition, the ACT matrix is used as a visual teaching tool to communicate ACT principles.^15^ Skills from mindfulness and acceptance-based therapies beyond acceptance and commitment therapy, such as dialectical behavior therapy (DBT),^16^ are incorporated as appropriate. Finally, in this case, clinical hypnosis was used to treat nausea and vomiting.

A range of techniques are used, but the goal is quite singular: to help patients tolerate the intense withdrawal symptoms so that the transition to buprenorphine-naloxone can be completed and they can move on to a new chapter of their lives, free from the suffering and limitations imposed by opioid dependency. Accordingly, ACT treatment puts personal values first, always emphasizing the kind of life and valued activities (e.g., quality time with family, meaningful work, etc.) the patient is moving toward by taking on this challenging transition. The goal is to dignify the distress and give it purpose and meaning.

Once the course has been set by clarifying personal values, the next priority is to help the patient identify, remember, and utilize his or her already-present effective strategies for getting through crises, as well as supplement the patient’s skills with helpful behavioral/psychological tools that round out his or her “coping toolbox.” To accomplish this, distress tolerance skills from dialectical behavior therapy are often useful. These skills include distraction, self-soothing, and mindful grounding techniques using sensory awareness (what you can see, what you can hear, what you can touch, etc.) that can help the person to “weather the storm” without making it worse by catastrophizing. C.D. was quite insightful about this, stating that she was keen not to engage in “awfulizing” and instead to engage in meaningful behaviors, such as going for walks, caring for her animals, and spending time with loved ones.

One of the most psychologically difficult aspects to bear of the transition to buprenorphine-naloxone is increased anxiety. We know that opioid withdrawal increases anxiety, and therefore it is useful to discuss with patients how to respond to this increased anxiety. Within mindfulness and acceptance-based frameworks such as ACT and DBT, we emphasize that the more one tries to suppress anxiety out of fear, the more it rebounds. In other words, fear of fear only leads to a negative cycle that becomes more and more overwhelming. We use metaphors to teach this principle (e.g., like the effort required to hold a beach ball under water). C.D. understood this immediately based on her prior experience living with pain, saying that “if you resist, it persists.” Using the ACT matrix, we highlighted the difference between “away moves” (behaviors driven by avoidance of pain and anxiety; e.g., lying in a dark under the covers, isolating) and “toward moves” (values-based approach behaviors; e.g., spending time with loved ones).

Finally, psychological intervention for C.D. involved clinical hypnosis for nausea and vomiting symptoms. C.D. was already familiar with hypnotherapy for pain relief^17^; however, she was not aware that it could be used for disorders of gut–brain interaction,^18^ such as cyclic vomiting syndrome. Indeed, although the published evidence for the Manchester gut-focused hypnotherapy protocol is primarily based on treatment of irritable bowel syndrome,^18,19^ the protocol also has been used successfully for cases of cyclic vomiting syndrome in Manchester (Pamela Cruikshanks, personal communication, September 2019). Accordingly, A.W. guided C.D. through a hypnotherapy exercise that induces deep relaxation targeted at the gut–brain axis. C.D. reported feeling “very, very calm” afterward. Creating a sense of psychological safety through hypnosis can reduce the likelihood of vomiting, as the brain processes many inputs, including psychological stress, perceived danger, and opioid withdrawal, which converge and summate to trigger the vomiting response. A.W. provided C.D. with a link to audio recordings she had made that could be accessed through the internet by her patients. These recordings included mindfulness and hypnotherapy exercises, which C.D. found useful and used repeatedly to help her to “ride the waves” of pain, nausea, and anxiety during this important transition. Notably, to date, A.W. and C.D. have not met in person, and all psychotherapy, mindfulness, and hypnotherapy sessions were conducted over the Ontario Telehealth Network or (when it failed likely due to a sudden surge in capacity during the initial COVID-19 crisis) by telephone.

Three weeks have elapsed since the day when the in-person induction of buprenorphine-naloxone took place. Currently C.D.’s nausea and vomiting have stabilized, and given her sensitivity to fluctuations in opioid dose, the decision was made to stay on 16 mg of buprenorphine-naloxone for some time. We have started to introduce neuropathic pain medications for the PHN. C.D. has a long road ahead and she will continue to receive the support of the TPS team. She is coming to terms with the fact that there is no “easy” solution or quick fix to her health problem.

Patients living with persistent pain and taking long-term opioids often require ever-increasing doses to maintain pain control due to opioid tolerance, while at the same time experiencing more and more side effects, including opioid-induced hyperalgesia.^20^ Our experience with buprenorphine-naloxone for complex, chronic, opioid-dependent patients with pain is that it can provide effective pain relief while not feeding into the spiral of escalating opioid requirements typically seen with μ-opioid agonists.^21^ From a harm reduction perspective (i.e., likelihood of opioid overdose), the safety profile of buprenorphine-naloxone is superior to that of methadone^22^; hence, we selectively opt for buprenorphine-naloxone preferentially over methadone for patients struggling with opioid escalation/management in the context of a chronic persistent pain.

## Case #2: Home Induction Protocol for Buprenorphine-naloxone during the COVID-19 Crisis in the Opioid Deprescribing Program, Calgary, AB

K.S. is a 44-year-old married male currently on disability, who was referred to the Opioid Deprescribing Program secondary to chronic opioid therapy, continued chronic pain, complex medical morbidities, and complex mental health morbidities. He attended an educational lecture on opioid deprescribing and was subsequently offered a consult. He presented for his initial consultation at the beginning of March 2020. He is supported by Assured Income for the Severely Handicapped and lives with his wife, parents, and autistic niece. He has struggled with chronic degenerative myofascial lumbar back pain, along with widespread pain, for which he was prescribed hydromorphone. He also described a right S1 radiculopathy and joint pain reported as 8–10/10 pain intensity daily on a numerical rating scale. He had been seen at the Spine Triage and Assessment Program at Foothills Medical Center where surgery was ruled out. Medical comorbidities included diabetes mellitus type 2, obstructive sleep apnea with CPAP, dyslipidemia, restless leg syndrome, and hypertension. Mental health diagnoses included a history of bipolar disorder. Nonopioid medications included moclobemide, lurasidone, pramipexole, lamotrigine, dextroamphetamine, rosuvastatin, mirabigron, semaglutide injection, diclofenac, ondansetron, gabapentin, and topical lidocaine/gabapentin/diclofenac. He is also prescribed cannabis oil in a 1:1 ratio of THC:CBD. Initial multidisciplinary assessment determined a borderline personality disorder and somatic symptom disorder, with predominant pain along with a high level of pain catastrophizing (scoring in the 85th percentile on the Pain Catastrophizing Scale).

His chronic opioid therapy included hydromorphone 8 mg q4 h (40 mg daily) and hydromorph contin 3 mg TID, a milligram morphine equivalent dose of 245 mg/day. He often ran out of his medications early, due to uncontrolled pain. There was no history of addiction and no aberrant use of his prescribed opioids, including crushing, snorting, injecting, or purchasing illicit or other prescribed opioids. At the initial visit, buprenorphine-naloxone was discussed as an alternative treatment if he was unable to taper his opioid dose and his pain could not be stabilized. A referral to interventional physiatry was also completed, but due to the COVID-19 pandemic it was put on temporary hold as the program was shut down. It was decided to start with an initial taper of 3 mg/day of his hydromoph contin dose, representing 6% of his total daily milligram morphine equivalent dose.

At 4 weeks, a telemedicine call was arranged for his next appointment instead of meeting in person. At the beginning of April 2020, the clinic made the decision to eliminate face-to-face appointments to reduce risk of viral transmission. The plan was to taper another 3 mg of hydromorph contin. K.S. reported a recent fall during which he injured a knee. He complained of increased pain and reported a consequent increase in opioid use, which depleted his medication supply early. K.S. admitted that the opioids were causing distress and impairment in his life. He was using more than intended in a day, unable to cut down or stop using them, craving them, especially when he was distressed, and they were affecting his mental health. He met the criteria for opioid use disorder, moderate and was unable to taper off his medication. After education and a discussion of the possible benefits of buprenorphine-naloxone, a first-line treatment for opioid use disorder,^23^ K.S. decided that it would be best for him. Education was provided on a home induction protocol and a prescription was called into the pharmacy.

On day 2 of the home induction, K.S. was called and reported feeling better and was stable at 18 mg of buprenorphine-naloxone. His mood had improved, his pain had significantly reduced, and he was thankful for his decision to initiate buprenorphine-naloxone. A follow-up via telephone was done 1 week later when K.S. reported that his pain intensity had dropped to a 2/10 and was not causing him any distress or impairment throughout the day. He did notice some pain at the end of the day, which led to cravings, but these would go away after a couple hours without any further intervention.

He subsequently met with a therapist via telemedicine within the Opioid Deprescribing Program and they reviewed cravings and triggers for him. DBT skills were reviewed with a focus on distress tolerance and a plan was implemented to continue one-on-one psychological support until he starts a DBT skills group. The goal of DBT in this context is to reduce emotional distress in order to help reduce pain and improve his ability to cope with the pain. Psychological distress and catastrophizing are known to increase pain,^24^ and an acceptance and values-based approach along with emotion regulation skills, as previously described, is of great importance in developing long-term and meaningful improvement in quality of life.^25^

### Home Induction

The home induction protocol for prescription opioids was designed to improve patient understanding and reduce patient discomfort and for educational purposes to improve physician prescribing. It is currently part of the continuing medical education for chronic pain offered by the Alberta Pain Society. One barrier to prescribing buprenorphine-naloxone involves a lack of knowledge and skill in the induction phase^26^; hence, developing a simplified protocol was important. In-office, monitored inductions have been the traditional way to initiate buprenorphine-naloxone treatment, but home inductions have similar efficacy and safety.^27–29^ In Canada there is a practice of daily witnessed dosing at the clinic or pharmacy for patients prescribed methadone or buprenorphine-naloxone, although this can lead to poorer retention to treatment and higher costs to the health care system with no known benefits^30,31^ compared to patients who are dispensed weekly/monthly medications. The use of the home induction protocol, which eliminates unnecessary face-to-face visits, is vital to reduce COVID-19 exposure and transmission in the setting of a pandemic.

**Figure 1.**
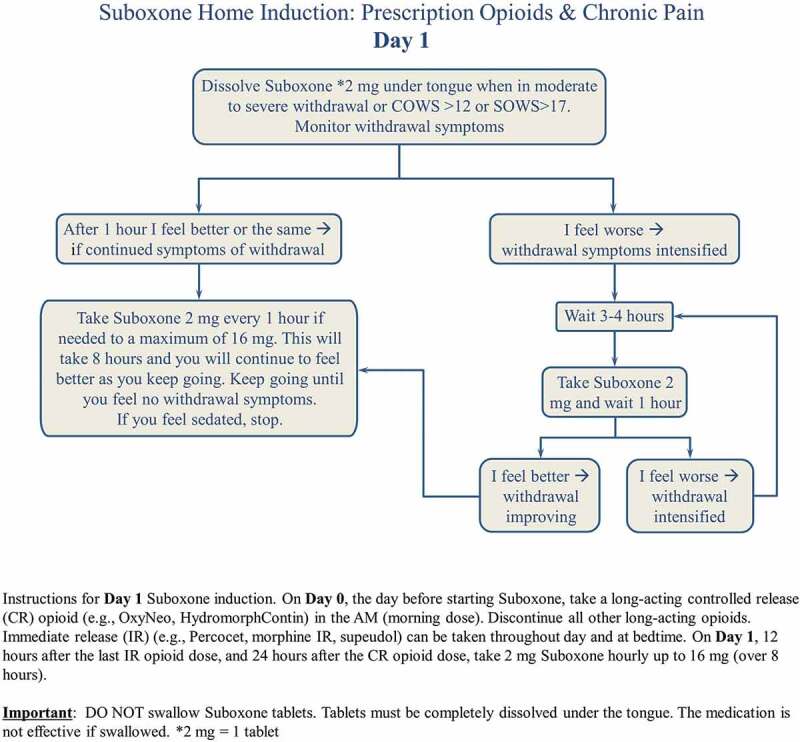
Suboxone Home Induction Day 1

**Figure 2.**
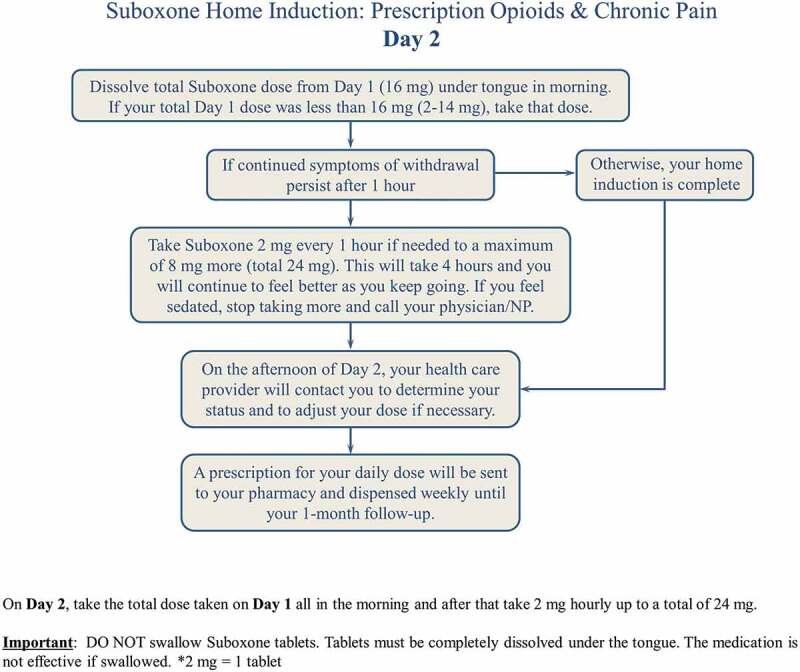
Suboxone Home Induction Day 2

The home induction protocol (Figure 1) begins on day 0, the morning before the start of the home induction. The patient stops the long-acting opioid the morning before the start of the home induction and continues taking the short-acting medications until the night before induction. The patient must take the last long-acting opioid dose 24 h before and the short acting dose 12 h before initiation of buprenorphine naloxone. The patient is advised to wait until he or she is in moderate withdrawal (defined by a Subjective Opioid Withdrawal Scale score >17).^32^ On day 1, a prescription is initiated for buprenorphine-naloxone 2 mg SL q1 h for 8 h (16 mg in total). The patient is advised to “take 2 mg 1 tablet every hour until you start to feel better and the pain improves up to a maximum of 16 mg.” On day 2 (Figure 2) the patient is prescribed two 8 mg tablets (16 mg) for the morning dose then an additional 2 mg q1 h for 4 h for a total daily dose of 24 mg. For patients whose day 1 dose was less than 16 mg, this dose (2–14 mg) becomes the daily total until they are re-assessed. The prescriber then calls the patient on day 2 in the afternoon to confirm the dose and subsequently sends the pharmacy a prescription for 28 days. Over the last 2 years, this protocol has been successfully implemented at the Opioid Deprescribing Program and modified for the Opioid Dependency Program for illicit fentanyl.

For patient K.S. about 30 min was spent discussing the buprenorphine-naloxone home induction. A handout was provided (Figures 1 and 2). On day 1, K.S. completed the maximum dose of 16 mg with no complications. On day 2, K.S. took the total from day 1 (16 mg) in the morning in one dose and then added a second dose of 2 mg 1 h later. He stopped at this dose. He was called by R.T. on day 2 and his dose was confirmed at 18 mg, and he was subsequently provided with a prescription for 28 days, with no witnessed dosing. This was all completed with telephone calls. An example of a prescription in order to complete the Buprenorphine-naloxone home induction  is presented in Appendix A.

### Technology and Clinical Care

During C.D.’s buprenorphine-naloxone induction, H.C. was, within minutes, able to assemble the entire team via teleconference. This was essential given the lack of face-to-face peer support during this time and essential with respect to sharing ideas and creating a swift plan. This was as helpful in quelling H.C.’s anxieties as it was to further developing the care plan for C.D. A.W.’s telehealth visits were immediately booked by our administrative assistant. C.D. had a home computer and was able to connect via the Ministry of Health and Long Term Care’s telehealth portal.

R.T. was consulted given his experience with buprenorphine-naloxone inductions and the numerous successful home inductions that he and his team have performed. Because C.D.’s rocky induction and cyclic vomiting was indeed novel to him, a larger discussion ensued during our teleconference with respect to home-based buprenorphine-naloxone inductions. R.T.’s team has smoothly transitioned patients from the comfort of their homes over the past years (i.e., case of K.S.). The travel that C.D. endured on the day of the induction (particularly on the way home while experiencing moderate opioid withdrawal) likely contributed to her visit to the emergency department, which could have been avoided.

A recent manuscript highlighted some of the benefits and challenges associated with the rapid introduction of telephone and e-health pain management services^33^ during COVID-19. Emergency departments are using telemedicine to triage patients with COVID-19 symptoms. In dealing with C.D.’s case, telephone, provincial telemedicine, and social media services were utilized. The Toronto General Hospital TPS clinic has already implemented a mobile health e-platform (Manage My Pain) into clinical care^34^ and is testing this solution. As e-health technology evolves, platforms that can enable physical examination would be preferable for new consultations. The case of K.S. makes it clear that successful home buprenorphine-naloxone inductions are feasible and likely represent a viable clinical pathway for the future given North America’s current opioid crisis.

## The TPS Psychology Team—Lessons Learned regarding Complex Chronic Pain and Buprenorphine-Naloxone Inductions

Patient education is paramount. Patients routinely receive educational information about the induction process, including a “road map” of the steps involved (see Figures 1 and 2) and frank discussion to manage expectations. The induction protocol is presented and explained to ensure that patients understand the process and their questions are answered satisfactorily. Over time and with experience, we have learned that it is important to screen for several psychological risk factors for complex chronic pain patients if the decision is made to pursue a buprenorphine-naloxone induction. The following are three clinical flags to denote that a patient may need a higher level of clinical support during the induction period, which can be a time of greater distress than baseline. However, patients with a history of other disorders (e.g., major depressive disorder, bipolar disorder, anxiety disorders) also require specialized care and monitoring during buprenorphine-naloxone induction and especially during home induction.

1. History of trauma/current PTSD. In our clinical experience, patients with a history of trauma are more likely to have difficulty with opioid withdrawal and the transition to buprenorphine-naloxone. The increased panic and anxiety during this time requires additional skills training to successfully tolerate and move beyond this transition; however, with support, patients can reach their treatment goals.
2. History of suicidal ideation and behavior. Patients in withdrawal from opioids may experience dysphoria. In patients with a history of suicidal behavior, a suicidal crisis can be triggered. Clinicians should screen in advance for this history (particularly for a history of high-lethality behaviors), monitor closely, and offer additional supports if needed. With psychological support, patients with this history can successfully transition to buprenorphine-suboxone.
3. Daily anxiolytic use. In our clinical experience, patients who are using both opioids and anxiolytics *daily* (not intermittent anxiolytic use but daily high-dose use) are more likely to have difficulty with the buprenorphine-naloxone transition. Acceptance and commitment therapy^35^ or motivational interviewing^36^ prior to attempting buprenorphine-naloxone induction may be of help in increasing motivation to tolerance distress, as well as skill development in acceptance of aversive sensations and distress tolerance for emotions.^21^

## The Opioid Deprescribing Team—Lessons Learned regarding Complex Pain and Buprenorphine/Naloxone Home Inductions

All patients who are enrolled in the program undergo an education session prior to their first appointment. This is important in discussing the role of opioid deprescribing to discontinuation and improvements in pain^37^ and that enrollment in the program is voluntary. The session reviews evidence and introduces the patients to the interdisciplinary team, including psychology, nursing, nurse practitioners, administration, and physicians. A small section includes the possibility of buprenorphine-naloxone for some patients, specifically those with addiction or suspected opioid-induced hyperalgesia^38,39^ and/or opioid antinociceptive tolerance.^40^

1. About 30% of patients presenting to tertiary chronic pain programs have a comorbidity of borderline personality disorder, leading to higher complexity for both the treatment of their pain and mental health.^41^ Having skilled therapists with training in DBT has been extremely helpful in improving psychological distress and hence pain outcomes. Often patients with high distress and high anxiety require significant reassurance, validation, and education prior to the buprenorphine-naloxone induction, similar to patients with a history of trauma/PTSD.
2. Education is key. Handouts and confirmation of the protocol and possible side effects are key. A video, forthcoming from Alberta Health Services, Addiction and Mental Health, will add extra education as well. The patient should be advised of proper administration of the medication. Phone calls on day 2 are extremely important to determine dose and alleviate anxiety.
3. Often, a difficulty in tolerating the medication stems from ingestion of the medication orally, instead of letting the medication be absorbed sublingually. This can lead to gastrointestinal upset, nausea, and vomiting. Larger tablets (i.e., an 8 mg SL tab) will take longer to dissolve (up to 10–15 min). If any portion of the tablet is orally ingested, the patient will have a higher chance of experiencing gastrointestingal side effects. When this is suspected, it may also be advised for the patient to rinse his or her mouth after dissolving the medication rather than swallowing the saliva, thereby minimizing oral ingestion.
4. It is important to discuss the risk of precipitated withdrawal with the patient, along with proper management of it. Precipitated withdrawal occurs when the patient starts buprenorphine-naloxone before sufficient opioid withdrawal is reached (i.e., there are still opioids in the system). The patient is thus advised to wait for the symptoms of precipitated withdrawal, which often presents as a rapid rise in withdrawal symptoms, to settle and for natural opioid withdrawal to occur, prior to continuing induction. This may take 3 to 4 h. Withdrawal symptoms may be alleviated during the process with the judicial use of medications such as clonidine and gabapentin.

## Conclusions

C.D. and K.S. both highlight complex chronic pain and opioid use disorder cases that were facilitated via telemedicine during the COVID-19 pandemic. In the case of C.D. it was an abrupt transition that, without the remote capabilities being given to the team to engage patients, may have ended with greater distress. The case of K.S. illustrates that with proper instructions by the team in advance of starting buprenorphine-naloxone, a home buprenorphine induction of a patient on high-dose opioids can be managed safely by the patient in the comfort of his or her own home. Telemedicine has been thrust to the forefront of health care in the past month. Based on our positive interactions with the team and patients and the ability to bring the team together to solve complex situations at the drop of a hat, it is hard to imagine returning to health care without the ability to remotely assess and interact with our patients post-COVID-19.

## Appendix A. Example of a home initiation prescription for buprenorphine-naloxone

Name: JOHN DOE

Date of issue: July 1, 2020

Day 1: Buprenorphine-naloxone 2 mg SL q1 h PRN, maximum 16 mg (8 tabs)

Day 2: Buprenorphine-naloxone 16 mg SL QAM (2 × 8 mg tabs) and 2 mg SL q1 h PRN, maximum 8 mg (4 tabs) for maximum total daily dose of 24 mg

Dispense 2 × 8 mg tabs and 12 × 2 mg tabs. Dispense all at once. No daily or witnessed dosing.

Note: Discontinue all other opioids. Advise patient to stop Immediate release (IR) opioid doses 12 h prior to first dose and Controlled release/Sustained release (CR/SR) doses 24 h prior to first dose.
